# Penile squamous cell carcinoma with posterior urethra involvement: A case report

**DOI:** 10.1016/j.radcr.2025.06.065

**Published:** 2025-07-23

**Authors:** Mahyar Daskareh, Elham Rahmanipour, Maryam Sadat Alamdar

**Affiliations:** a Department of Radiology, Ziaeian Hospital, Tehran University of Medical Sciences, Tehran, Iran; b Department of Radiology, University of California San Diego, San Diego, CA, USA; c Immunology Research Center, Mashhad University of Medical Sciences, Mashhad, Iran; d Nuclear Medicine Research Center, Ghaem Hospital, Mashhad University of Medical Sciences, Mashhad, Iran

**Keywords:** Magnetic resonance imaging (MRI), Squamous cell carcinoma (SCC), Human papillomavirus (HPV), Penile tumor

## Abstract

Penile squamous cell carcinoma (SCC) is a rare malignancy that is more prevalent among immunocompromised individuals. Given that the tumor primarily originates along the penile shaft, it typically affects the anterior urethra. In contrast, primary urethral cancers (PUC) more commonly involve the posterior urethra. The imaging features of penile SCC and PUC often overlap, making the pattern of urethral infiltration a key factor in distinguishing between these 2 conditions. Magnetic resonance imaging (MRI) plays a crucial role in local staging and in identifying the patterns of urethral involvement. We present the case of a 40-year-old man diagnosed with penile SCC following a kidney transplant and prolonged immunosuppressive therapy. This case illustrates the rare occurrence of penile SCC with posterior urethral involvement, as demonstrated through MRI findings. This case sheds light on the possible atypical penile SCC urethral involvement. We aim to highlight its significance for clinicians and radiologists regarding the absence of similar diagnostic imaging illustrations of penile SCC with such a posterior urethra infiltration.

## Graphical Abstract

**Figure:**
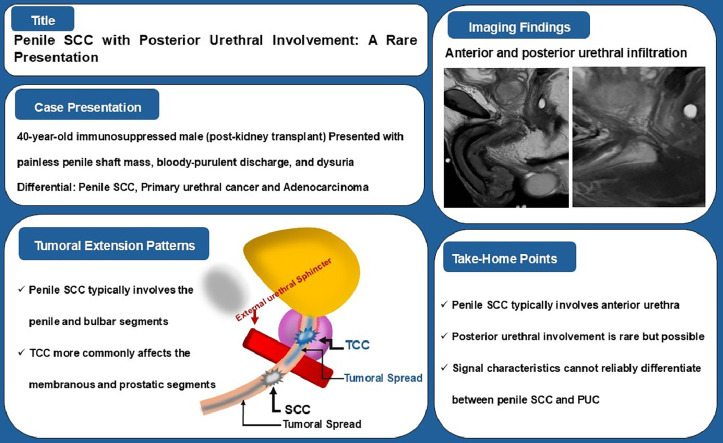

## Introduction

Primary penile tumors are rare, affecting fewer than 1 per 100,000 males in the US [1], typically older men averaging 60 years of age [2]. However, young immunosuppressed patients show a dramatically increased incidence, about 17 times higher than the general population [3]. Increased susceptibility to human papillomavirus (HPV), found in nearly 48% of penile cancers, explains this phenomenon [[3], [4], [5]]. Penile and primary urethral cancers (PUC) demonstrate similar imaging findings, differentiated mainly by urethral infiltration patterns [4]. Squamous cell carcinoma (SCC) accounts for over 95% of penile tumors but only 20% of PUCs [6].

The main question a radiologist should answer is what urethral segments are involved [7]. Penile SCC typically involves the penile and bulbar urethra, while PUC primarily affects the prostatic and membranous urethra [8]. MRI significantly aids local staging and assessment of urethral involvement [4], but sometimes the tumor extends beyond typical behavior. This report presents an unusual case of penile SCC with atypical posterior urethral infiltration.

## Case report

A 40-year-old male kidney transplant recipient presented with a progressively enlarging penile mass. The transplant, conducted 5 years prior due to cryptogenic renal failure, necessitated ongoing immunosuppression with mycophenolate mofetil (1 g orally twice daily) and cyclosporine (15 mg/kg/day orally, tapered to a maintenance dose of 5-10 mg/kg/day divided into 2 doses), as per the Kidney Disease Improving Global Outcomes (KDIGO) guidelines. His baseline serum creatinine was 2.5 mg/dL. He reported a painless penile lesion for 2 months, accompanied by urinary difficulty and bloody discharge, without systemic symptoms or prior urogenital history. There was no family history of urogenital malignancy.

**Fig. 1:**
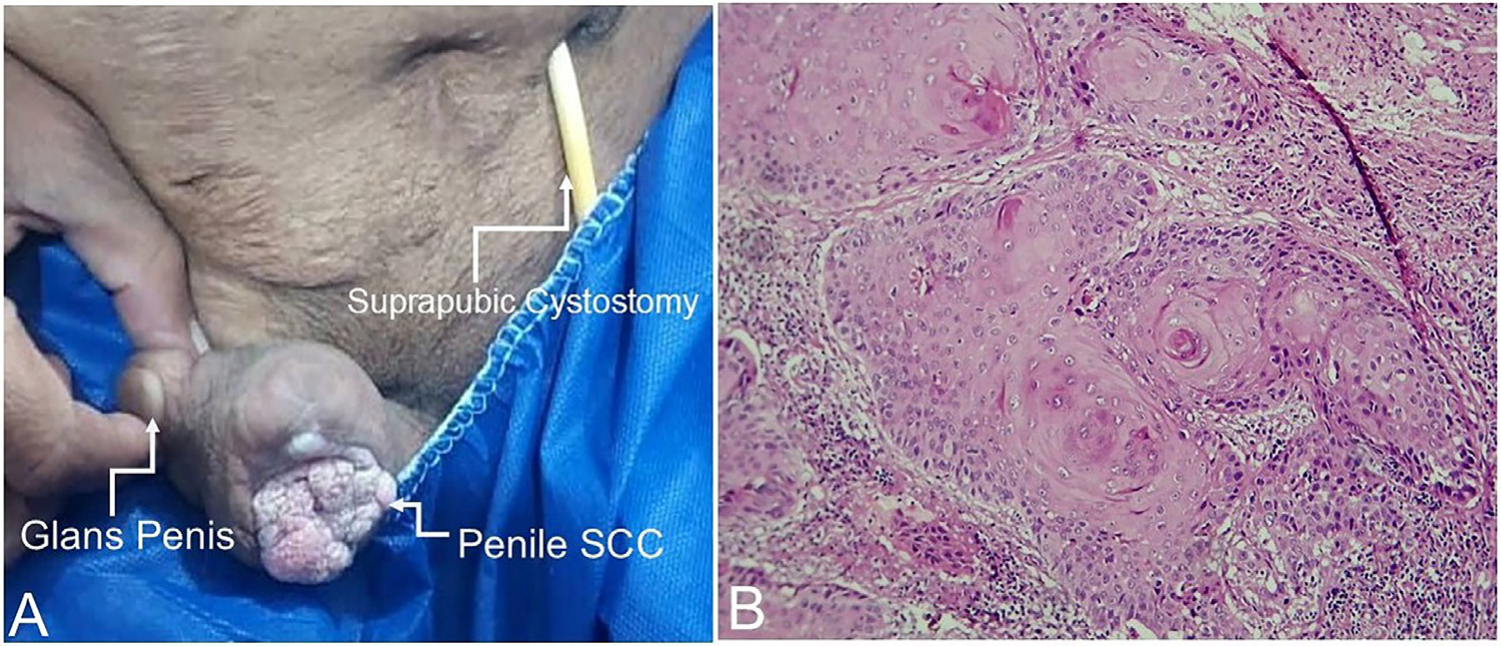
Clinical photograph (A) showing a fungating exophytic mass consistent with penile squamous cell carcinoma (SCC) involving the distal penile shaft and glans penis, with a suprapubic cystostomy catheter in place for urinary diversion. Histopathological image (B) (H&E stain) demonstrating invasive keratinizing squamous cell carcinoma with prominent keratin pearls.

Examination revealed a 46 × 28 mm polypoid mass on the ventral surface and distal portion of the penile shaft with scant discharge. There were no palpable lymph nodes or constitutional symptoms (Fig. 1A). Laboratory evaluation indicated renal dysfunction and anemia (Table 1). Differential diagnoses included penile SCC, lymphoma, adenocarcinoma, and PUC. Biopsy confirmed undifferentiated keratinizing SCC (Fig. 1B), and HPV subtype 16 via PCR and in situ hybridization.

**Table 1:** Laboratory findings.

Parameter	Patient value	Reference range
Blood urea nitrogen (BUN)	35 mg/dL	7-20 mg/dL
Serum creatinine	2.7 mg/dL	0.6-1.3 mg/dL
Sodium	139 mmol/L	135-145 mmol/L
Potassium	3.6 mmol/L	3.5-5.0 mmol/L
Calcium	7.9 mg/dL	8.5-10.2 mg/dL
Albumin	3.2 g/dL	3.5-5.5 g/dL
Hemoglobin (Hb)	7.4 g/dL	13.5-17.5 g/dL
White blood cell count	7.2 × 10³/µL	4.0-10.5 × 10³/µL
Platelet count	164 × 10³/µL	150-400 × 10³/µL

**Fig. 2:**
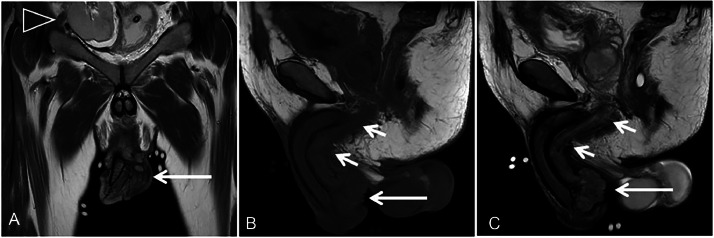
T2W coronal image (A) shows a large mass (long arrow) along the ventral aspect of the penile shaft; the transplanted kidney is visible in the right pelvic cavity (arrowhead). T1W sagittal image (B) demonstrates a homogeneously isointense mass (long arrow) infiltrating the bulbar and penile urethrae (short arrows). T2W sagittal image (C) reveals heterogeneous high signal intensity in the same urethral regions (short arrows), indicating tumor infiltration.

**Fig. 3:**
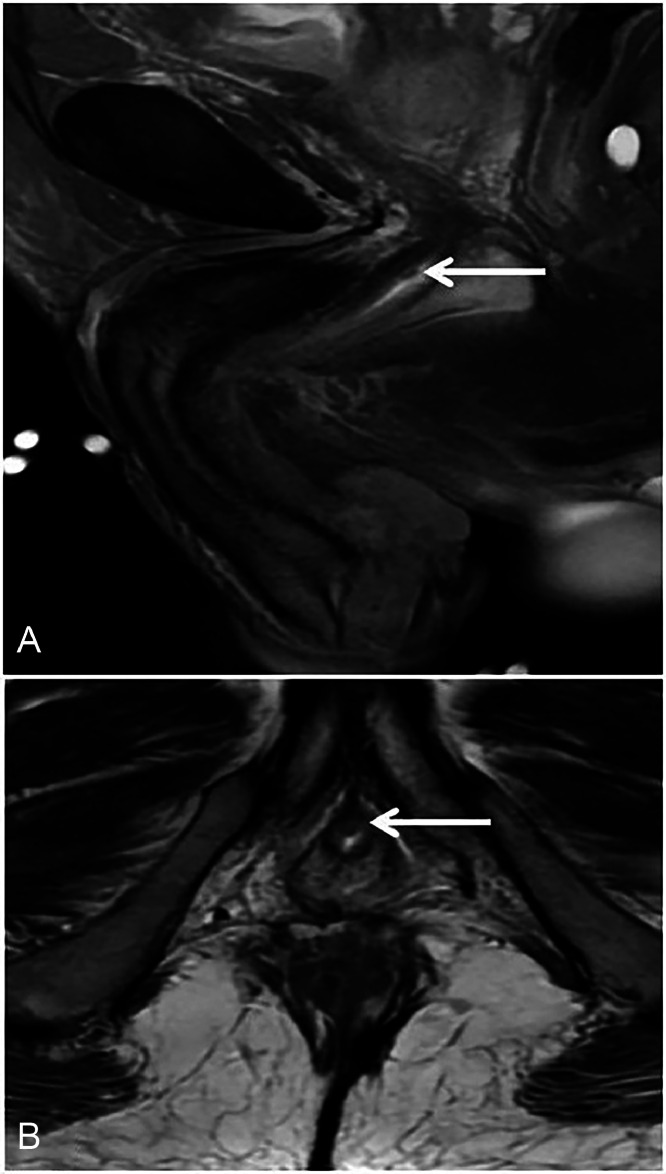
Sagittal T2W fat-saturated image (A) shows an intraluminal nodule (arrow) within the membranous segment of the urethra, suggestive of tumoral infiltration. Axial T2W image (B) at the corresponding level demonstrates a low-signal intraluminal nodule (arrow), consistent with tumor involvement of the membranous urethra.

Un-enhanced penile MRI (Fig. 2) revealed a polypoid intermediate signal mass on the ventral surface of the penile shaft, infiltrating fossa navicularis, penile urethra, and corpus spongiosum, sparing the corpora cavernosa. Notably, tumor infiltration into the membranous and bulbar urethra (Fig. 3) challenged the typical anterior-only urethral involvement expected with penile SCC. Discriminating penile/ urethral tumors via MRI alone remains complex, and even histopathology may not always provide definitive differentiation [4]. Pelvic and abdominal imaging showed no nodal or distant metastasis.

Given immunosuppression and advanced tumor stage, the patient underwent suprapubic cystostomy and initiated chemotherapy with 5-fluorouracil and mitomycin C, omitting radiotherapy due to the pelvic kidney graft.

## Discussion

This case presents a patient with penile SCC with an unusual posterior urethral extension. Although urethral involvement influences treatment planning, it is no longer a determinant of staging [9]. In our case, the tumor was classified as T2 due to corpus spongiosum infiltration. Malignant tumors with squamous or transitional histology will typically exhibit a relatively low signal on T1-weighted imaging (T1WI) and low to intermediate signal intensity on T2-weighted imaging (T2WI), with a heterogeneous pattern of post-contrast enhancement. Adenocarcinomas, however, exhibit relatively high signal on T2WI with variable enhancement [7].

On diffusion-weighted imaging (DWI) MRI, urethral tumors show distinct qualitative patterns. TCC appears with a high signal and moderate restriction, often extending from the bladder. SCC shows strong, uniform restriction and tends to invade anteriorly. Adenocarcinomas exhibit variable, sometimes heterogeneous signals, which can make them more challenging to distinguish. Lymphomas show uniformly high signals and appear homogeneously without necrosis. Prostate cancer typically presents as a contiguous urethral diffusion restriction, mainly involving the apex and membranous urethra due to their proximity to tumor spread [7,10].

Therefore, differentiation of tumoral histology solely based on the MRI signal is unreliable [11]. The urethra in males gender is lined by transitional cells in its prostatic and membranous portions and stratified squamous epithelium in the bulbous and penile portions [12]. As a result, transitional cell carcinomas consist of the major histological type of posterior urethral tumors, while tumors with anterior urethral extension are mainly SCC [13]. The present case report demonstrates that penile SCC would be able to extend beyond the expected anatomical boundaries with posterior urethral involvement. While MRI is a valuable imaging tool in determining tumor invasion into adjacent structures, such as the tunica albuginea and corpus spongiosum, it may lead us to the wrong histological subtype if we only consider the pattern of urethral disease [11].

Penile cancer in transplant recipients poses significant diagnostic and therapeutic challenges due to immunosuppressive treatment, which increases the risk of solid organ malignancies. Specifically, penile cancer incidence is elevated (standardized incidence ratio (SIR) of 4.13; 95% CI, 1.66-8.49) compared to the general population. Other frequent cancers include nonmelanoma skin cancer (SIR, 13.85), Kaposi sarcoma (SIR, 61.46), and kidney cancer (SIR, 6.66). Tumors in these patients often present aggressively and at advanced stages. Immunosuppression may mask symptoms, delaying diagnosis and complicating treatment due to the need to balance oncologic management with maintaining graft function [14], highlighting the need for increased awareness, routine screening, and early biopsies [15].

Management involves a balance between effective oncological therapies and ongoing immunosuppression to prevent graft rejection. Penectomy, chemotherapy, and radiotherapy may be undertaken, but patients are at higher risk of complications, including wound infection, lymphocele, and lymphedema. Immunotherapy, specifically immune checkpoint inhibitors like pembrolizumab, may be less effective in these immunocompromised individuals due to suppressed immune responses [16].

Finally, although this patient did not exhibit nodal metastases, lymph node involvement remains the most significant prognostic factor in penile SCC [17]. Patients with nodal metastasis exhibit significantly worse outcomes, with 5-year cancer-specific survival rates dropping from 85% to 100% in cases without inguinal involvement to significantly lower rates when pelvic or multiple nodal metastases are present [18].

## Conclusion

MRI is the preferred modality for assessing penile and urethral tumors, offering superior tissue characterization and precise delineation of extension. While signal characteristics aid differential diagnosis, anatomical localization is more diagnostically conclusive. However, infiltrative penile SCC may disregard anatomical-histological compartments, complicating staging and management.

## Patient consent

Written informed consent for the publication of this case report, including clinical details and images, was obtained from the patient. The consent form is retained by the authors.
